# Os-Artificial Vindicated

**Published:** 1870-02

**Authors:** W. F. Morrill


### DENTAL REGISTER.

Vol. XXIV.] FEBRUARY, 1870. [No. 2.

### Original Communications.

### OS-ARTIFICIAL VINDICATED.

BY W. F. MORRILL.

In the October Register I committed the indiscretion of commenting favorably on os-artificial as a proper protection over exposed pulps. The last Number of the Register contains a rejoinder to those remarks unhappily made. In fact, the "Miscellanies" contributor yields not the palm of victory, but adheres to his preconceived notions, in opposition to the claims of this important agent. Neither does he hesitate at wholesome reproof nor statement of facts. The few pebbles I had occasion then to drop into his crop, though not intended to impart august nutriment to his nature or understanding, yet were intended, by their intestinal concretions, to serve some obscure purpose of aiding his moral and intellectual digestion, all of which, I am pained to say, failed in its object. Had the case been otherwise, I should have been spared the necessity of furnishing, as I shall now, the records and testimony in support of one of the most valuable agents yet introduced into the practice of Dentistry.

We seek to know the truth. Opinions sustained by an array of facts carry weight. Extraneous or irrelevant matter not pertinent to the subject proper would scarcely be in good taste, besides burdening the reader; hence I shall endeavor to adduce further proof in favor of os-artificial, for the purpose herein named, without regard to other matters. The field of Dentistry is a rare one for the display of great gifts, such as the powers of observation, and to foresee results. Some men in Dental practice have wonderful facilities of quickly comprehending the pathological conditions of a case, and providing for the same. These perceptive. penetrating faculties make one operator excel another in practice. Some practitioners, having years of experience, and who have in their manipulations exposed thousands of pulps, virtually ignore all treatment. They act upon the maxim that "dead men tell no tales." The cudgel of extermination, twin relic of barbarism, has long had its advocates among those who venerate ancestral customs and habits. It is the devout wish that these excisions, arsenic devitalizers may ere long come to grief. It is high time now.

This little throbbing customer, denominated pulp, subject to a variety of moods and freaks, is a source of many woes. Habit and tradition have long affixed death as the penalty for its crimes. "If it were done, it were well it were done quickly;" but the lingering mode of poisoning, and the attendant train of evils resulting therefrom, ought to quite put to blush the murderer. The funeral obsequies, from the disintegrating moments, are too familiar for narration. Thenceforth the dead corpse hangs on its socket as a memorial at which coming generations may gaze, with mingled admiration and pride, as a specimen of the wonders and achievements of Dental art and science.

The introduction of os-artificial as a protection over exposed nerves or pulps, was, if I mistake not, brought to notice by the venerable Dr. Keep, of Boston. Prior to this, it had been known publicly as a material proper for temporary fillings, and a protection over their dentine partitions, proximating the exposed pulps. The excellence, for this latter purpose, was communicated to the professional friends of Dr. Keep, and it came rapidly into notoriety. Of course, like every thing else, it found friends and enemies ; that the former outnumber the latter, there is abundant reason for believing. Employing it in a considerable number of cases, in my own practice, I can record no more than one-half dozen failures, and these, probably, were owing to too much haste in the treatment. In several instances, cases while undergoing treatment have been examined after several weeks and months elapsed, since introducing the os-artificial, and the final filling delayed; and in each case the pulps were found preserved. If the pathological conditions and the manipulative skill are well understood, it is pretty difficult to see how failures can result. "The philosophy of filling teeth," says one greater than I, "is based on the unchangeableness of the conditions." First, if the conditions are diseased, they must be brought into a state of health ; the pathological conditions must be comprehended, and all other attendant circumstances connected with each case must be carefully noted. Many exposed pulps are lost by neglecting these important features.

But my own opinions in regard to the advantages of this material, are of small consequence beside those whose names and deeds are co-extensive wherever Dentistry is known. Let me here introduce their testimony.

Dr. Atkinson, a man of noble gifts, says : "He is held responsible who willfully destroys the pulps ; use those substances capable of producing an insoluable coagulum of the main tissues of the pulp, causing the healing of the exposed parts, when the filling of the tooth is a small work. To do this, put a wave of creosote over the exposed point of the pulp, until the coagulum of the structure is caused; then work it clean with tincture of callendula and water; dry out with bibulous paper, and have some os-artificial prepared. Let it be conveyed to the exposed part by capillary attraction, perhaps aided by tapping it lightly. When hard, fill out the remaining part of the cavity."

Now what is the result of this treatment? He says: "We apply the grandchild oxygen, viz., creosote C. H. O., which marries its second cousin albumen, C. H. N. O., of the tissues, thus securing a good neighborly feeling in the disturbed territory; and now to build a house that they may in harmony dwell, we bring in an Ishmaelite (os-artificial), to hedge in these thin skinned people from too near contact with the outside barbarian, or stranger, gold, with which we complete the outer wall, and from thermal shocks."

Dr. Wetherbee, among Boston's pride as an operator, says: "I believe that it is perfectly practicable to apply os-artificial to exposed pulp, even where decay has advanced so far as in excavating the cavity the pulp is made to bleed."

Dr. Francis, of New York city, a gentleman of fine attainments, says, in a recent essay: "I have removed fillings over exposed pulps capped with os-artificial some twelve months ago, and found a beautiful deposite of secondary dentine in each case, covering a healthy pulp, as proper tests indicated."

Prof. Taft, whose achievements as an operator are unquestioned, says: "When there are living pulps, the ease and facility thus afforded (using os-artificial) in introducing the filling afterwards, without any possibility of injuring the pulp, are among its advantages.

Dr. Salmon, than whom there is no more beautiful and ingenious operator, says he has used os-artificial for a considerable time, and suggests the use of a linen patch in contact with the exposed pulp, as possessing some advantages.

To multiply additional testimony seems an idle task. As well might a person dispute the advantages of brick and mortar for building purposes. Until it shall be demonstrated clearly that this material is productive of harm, and its merits not what are claimed for it, os-artificial must stand as the best protective for this purpose hitherto discovered. I have no disposition to quarrel or controvert with those who may think differently with respect to this agent; but it has a right to be adjudged fairly, and its exact virtues made apparent to those capable of investigating and appreciating them. Its strength of advantages can not be weakened by discussion of its merits.

